# Autologous Blood Injection Therapy for tendinopathy

**DOI:** 10.1186/1757-1146-3-S1-P11

**Published:** 2010-12-20

**Authors:** Olga Frankowski

**Affiliations:** 1 University of Salford, Salford, Greater Manchester, UK

## Introduction

The aim of this poster is to provide a rationale and critical overview of ‘Autologous Blood Injection Therapy’ a new and possibly superior treatment modality in the management of tendinopathy. The theoretical background will be appraised including its clinical application and perceived clinical effectiveness.

## Methods

literature review and critical appraisal of papers

## Results

Autologous blood injection therapy has been shown to have a significant positive effect on both pain and joint function, with concomitant therapies not being demonstrated to be required in the management of tendinopathy. However, the majority of studies were representative of patients being treated for refractory epicondylitis with different procedure techniques being employed. No studies compared blood re-injection therapy with dry needling alone, with minimal data available from controlled studies preventing a placebo response from being ruled out.

## Discussion

With current evidence on the efficacy of autologous blood injection therapy in the management of tendinopathy being insufficient in quantity and quality, the current National Institute for Health and Clinical Excellence guidelines state this procedure is to only be used with special arrangement. Future research should focus on randomised control trials that clearly describe previous or adjunctive therapeutic interventions with a minimum follow up of 1 year.

